# Modeling the influence of vitamin D deficiency on cigarette smoke-induced emphysema

**DOI:** 10.3389/fphys.2013.00132

**Published:** 2013-06-12

**Authors:** Mardi A. Crane-Godreau, Candice C. Black, Andrew J. Giustini, Tenzin Dechen, Jihan Ryu, James A. Jukosky, Hong-Kee Lee, Katherine Bessette, Nora R. Ratcliffe, P. Jack Hoopes, Steven Fiering, John A. Kelly, J. C. Leiter

**Affiliations:** ^1^Department of Microbiology and Immunology, Geisel School of Medicine at DartmouthLebanon, NH, USA; ^2^Veteran's Administration Research Facility, White River JunctionVT, USA; ^3^Department of Pathology, Geisel School of Medicine at DartmouthLebanon, NH, USA; ^4^Thayer School of Engineering, Dartmouth CollegeHanover, NH, USA; ^5^Department of Surgery and Radiation Oncology, Geisel School of Medicine at DartmouthLebanon, NH, USA; ^6^Department of Medicine, Geisel School of Medicine at DartmouthLebanon, NH, USA; ^7^Department of Physiology and Neuroscience, Geisel School of Medicine at DartmouthLebanon, NH, USA

**Keywords:** emphysema, vitamin D deficiency, second hand cigarette smoke, matrix metalloproteinase-9, tissue inhibitor of metalloproteinase-1, alpha-1 antitrypsin, chronic obstructive pulmonary disease, Teague smoke exposure device

## Abstract

Chronic obstructive pulmonary disease (COPD) is a major cause of morbidity and mortality worldwide. While the primary risk factor for COPD is cigarette smoke exposure, vitamin D deficiency has been epidemiologically implicated as a factor in the progressive development of COPD-associated emphysema. Because of difficulties inherent to studies involving multiple risk factors in the progression of COPD in humans, we developed a murine model in which to study the separate and combined effects of vitamin D deficiency and cigarette smoke exposure. During a 16-week period, mice were exposed to one of four conditions, control diet breathing room air (CD-NS), control diet with cigarette smoke exposure (CD-CSE), vitamin D deficient diet breathing room air (VDD-NS) or vitamin D deficient diet with cigarette smoke exposure (VDD-CSE). At the end of the exposure period, the lungs were examined by a pathologist and separately by morphometric analysis. In parallel experiments, mice were anesthetized for pulmonary function testing followed by sacrifice and analysis. Emphysema (determined by an increase in alveolar mean linear intercept length) was more severe in the VDD-CSE mice compared to control animals and animals exposed to VDD or CSE alone. The VDD-CSE and the CD-CSE mice had increased total lung capacity and increased static lung compliance. There was also a significant increase in the matrix metalloproteinase-9: tissue inhibitor of metalloproteinases-1 (TIMP-1) ratio in VDD-CSE mice compared with all controls. Alpha-1 antitrypsin (A1AT) expression was reduced in VDD-CSE mice as well. In summary, vitamin D deficiency, when combined with cigarette smoke exposure, seemed to accelerate the appearance of emphysemas, perhaps by virtue of an increased protease-antiprotease ratio in the combined VDD-CSE animals. These results support the value of our mouse model in the study of COPD.

## Introduction

Chronic obstructive pulmonary disease (COPD) is the third leading cause of chronic morbidity and mortality in the United States and is projected to rank fifth in 2020 in burden of disease worldwide (Rabe et al., 2007). Smoking, the main risk factor for COPD (Stockley et al., 2009), is a powerful inducer of inflammatory mediators, including oxidants and proteases (Spurzem and Rennard, 2005). The increased activity of proteases is important given that protease-antiprotease imbalance plays a key pathogenic role in the development of emphysema in COPD. In this regard, alpha-1-antitrypsin (A1AT) is the prototypical member of the serine protease inhibitor (serpin) superfamily of proteins, which have a major role in inactivating neutrophil elastase and other proteases to maintain protease-antiprotease balance (Lomas and Mahadeva, 2002). Alpha-1 antitrypsin deficiency (AATD), a genetic disease resulting in low levels of antiproteases, is a risk factor for emphysema, which supports the importance of protease-antiprotease balance in the pathogenesis of emphysema (Sandhaus, 2010). Another family of proteolytic enzymes, the matrix metalloproteinases (MMPs), and their inhibitors, tissue inhibitor of metalloproteinases (TIMPs), are involved in remodeling the extracellular matrix and in host defense (Elkington and Friedland, 2006) and are also associated with tissue destruction in emphysema (Abboud and Vimalanathan, 2008).

Although smoking and age are major determinants of lung function (as measured by the forced expiratory volume in 1 s; FEV1), there is an epidemiologic association between low serum concentrations of 25-hydroxy vitamin D and lung function (Black and Scragg, 2005). There is also an association between disease severity and genetic variability in vitamin D pathway genes found in cross-sectional studies of patients with COPD (Janssens et al., 2010). Indeed, there is increasing evidence for diverse roles for vitamin D, such as innate immunity and muscle strength that may contribute to respiratory health (Hughes and Norton, 2009). In a recent single center, double-blind study using high dose vitamin D supplementation, patients who entered the study with severe vitamin D deficiency, experienced fewer COPD exacerbation events after vitamin D supplementation (Lehouck et al., 2012). The results of this interventional study contrast with another study in which there were no differences in baseline vitamin D levels between smokers with rapid vs. slow declines in lung function over approximately 6 years of prospective follow-up (Kunisaki et al., 2011). This non-interventional study was limited—only one vitamin D measurement was made at the start of the study period. Thus, nothing is known about vitamin D intake or levels during the subsequent period of disease progression. Although there is increasing interest in vitamin D and lung disease, two important questions remain unanswered. First, can vitamin D deficiency act as a contributing risk factor for emphysema? Second, what are the mechanisms whereby reduced levels of vitamin D could directly influence the pathogenesis of emphysema? Putative mechanisms include immune modulation and lung tissue remodeling (Janssens et al., 2009).

Given the multitude of confounding factors, it is difficult to address any potential cooperative link between vitamin D deficiency and susceptibility to lung injury by cigarette smoke in humans. Therefore, we developed an animal model to study the separate and combined effects of vitamin D deficiency and cigarette smoke exposure on lung structure and function. We believe that this model provides a useful platform to investigate the individual and joint mechanisms that contribute to complex lung disorders.

## Materials and methods

### Mice, diet, and smoke-exposure

All aspects of the mouse protocol were approved by the Dartmouth Institutional Animal Care and Use Committee and/or the White River Junction VA Institutional Animal Care and Use Committee (*IACUC*). All mice were purchased from the National Cancer Institute at 5–6 weeks of age. Mice were housed with 12-hour dark and light cycles and given free access to food and water. Mice were weighed weekly and monitored for changes in health status. In early experiments, cigarette smoke exposed male mice became aggressive and were excluded from study for that reason. Therefore, we studied female FVB mice, a strain used in cigarette exposure experiments (Keith et al., 2004; Siddens et al., 2012). The animals were fed either a vitamin D deficient diet (VDD) or a vitamin D replete diet as a control (CD) (Harlan-Teklad, Madison, WI, USA; VDD number TD.09498 and CD number TD.09499) beginning at 6 weeks of age. The vitamin D replete diet contained 2.2 i.u vitamin D3/gm of food. Assuming mice consume ~3 gms of food per day, the control diet animals received near the upper limit of the acceptable daily dose of vitamin D recommended by the Endocrine Society. Because of the effect of vitamin D on lung structure in developing mice (Zosky et al., 2011), we started dietary restriction at six weeks of age when development has progressed beyond the alveolar stage of lung growth (Backstrom et al., 2011). Six weeks after beginning each diet, mice were exposed to a combination of second hand cigarette smoke (89%) and primary cigarette smoke (11%) produced in a Teague-TE10 smoke exposure device (Griffith and Hancock, 1985) (Teague Enterprises, Woodland, CA), using Research Cigarettes 3R4F (University of Kentucky College of Agriculture Reference Cigarette Program, Lexington, KY), for 4 h/day, 5 days/week, for 16 weeks. The average Total Suspended Particle (TSP) content within the chamber was 75 mg/m^3^. Control mice breathed filtered air and had handling similar to smoke-exposed mice. Chronic smoke exposures were conducted in four different experiments each with 48 mice randomly divided into the four treatment groups.

Vitamin D levels were confirmed either by Enzyme Immunoassay (EIA) according to the manufacturer's instructions (Immunodiagnostic Systems Ltd., Fountain Hills, AZ) or by outside laboratories (Dartmouth Reference Laboratory, Lebanon NH or ZRT Laboratory, Beaverton, OR). We sent samples to two outside laboratories to confirm our initial findings. Results from the two labs were consistent.

We studied the effects of two interventions, vitamin D deficiency and cigarette smoke exposure. Mice were randomly assigned to one of four treatment groups; control diet-non smoke exposed (CD-NS), control diet-cigarette smoke exposed (CD-CSE), vitamin D deficient-non smoke exposed (VDD-NS) and vitamin D deficient-cigarette smoke exposed (VDD-CSE). The results of this study were analyzed with a Two-Way ANOVA design in which each treatment (cigarette smoke exposure and vitamin D-deficiency) was a between subjects factor. When the ANOVA indicated that significant differences existed among treatment groups, specific pre-planned comparisons were made using *P*-values adjusted by the Bonferroni method. Data were reported as the mean ± the standard error of the mean (SEM).

### Respiratory mechanics

For pulmonary function testing, each mouse was anesthetized by intraperitoneal (i.p.) injection of Xylazine hydrochloride (10 mg/kg) and pentobarbital sodium (50 mg/kg). Tracheostomy was performed using a 19-gauge cannula and secured with suture ties. Animals were mechanically ventilated with a tidal volume of 10 ml/kg at a frequency of 150 breaths/min using a computer-controlled volume ventilator (*flexi*Vent™, SCIREQ, Montreal, PQ) against a positive end-expiratory pressure (PEEP) of 3 cm H_2_O. To facilitate measurements of respiratory mechanics, anesthetized animals were paralyzed with pancuronium bromide (0.5 mg/kg i.p.). To ensure adequate anesthesia, heart rate was monitored with a continuous electrocardiogram tracing.

The *flexi*Vent™ system uses a forced oscillation method to create pressure-volume curves in anesthetized animals to measure respiratory system mechanics (Bates and Suki, 2008; Vanoirbeek et al., 2010). The measured variables were fitted to a variety of models depending on the particular stimulus, and the measures of respiratory mechanics were derived from the parameters in each model. For example, the Snapshot-150 perturbation used a fixed frequency (2.5 Hz) pressure oscillation applied at the trachea, and measurements of respiratory system resistance [R], and compliance [C] were derived from these pressure oscillations using a single compartment model with an equation of motion dependent only on flow and volume. The Quick-prime-3 perturbation consisted of a multiple frequency pressure oscillation used to calculate the input impedance of the respiratory system. From the impedance measurement, estimates of airway resistance [Rn], tissue damping [G], and tissue elasticity [H] were made using a constant phase model. Finally, pressure-volume curves were used to measure static compliance [Cst], total lung capacity [TLC], and hysteresis [Area]. All of the fitted parameters were calculated using *flexi*Vent™ software. The *flexi*Vent™ system has been widely used to make measurements of respiratory mechanics in mice and has been validated in chronic measurements of respiratory mechanics in mice by comparing measurements derived from the *flexi*Vent™ system to more traditional measurements of respiratory mechanics (De Vleeschauwer et al., 2011).

### Lung pathology

To preclude the possibility of confounding effects from the measurements of respiratory mechanics, the analysis of lung pathology, including morphometric data and images, came from mice that were not studied by *flexi*Vent™, and alternate groups of mice were selected for analysis of lung pathology or lung mechanics. Mice selected for analysis of lung pathology were euthanized by cardiac puncture to avoid the potential effects of CO_2_ asphyxiation on atelectasis. Lungs were inflated at 20 cm H_2_O pressure with a 2.5% gluteraldehyde solution. Blocks of fixed tissue were excised, placed in cassettes and set in paraffin. Hematoxylin and eosin stained slides were examined by light microscopy with 4×, 10× and 20× magnification with a standard Olympus microscope by a board certified surgical pathologist.

The pathologist was blinded to the treatment group of each animal. The presence/absence and degree of emphysema, acute neutrophilic inflammation of the tubular airways (bronchi and bronchioles), chronic (mainly lymphocytic) inflammation of the tubular airways, chronic parenchymal inflammation (of spongy alveolar tissue), pigmented macrophages within tubular airways, and pneumocyte cell hyperplasia (without further interpretation as pre-neoplastic or reactive) was recorded for each mouse lung. A four tiered score (0, +, ++, +++) was recorded for each of the morphological features. Zero, meaning the finding was absent in the lung tissue on the H&E slide, +, meaning that the finding was focal (limited to either one tubular airway or 1 high power field (HPF) of parenchyma), ++, meaning the finding was present in two tubular airways or 2 separate HPF's or parenchyma, and +++, meaning the finding was present in 3 or more tubular airways or HPFs. Pigmented macrophages, which are commonly seen in human smokers and called “smokers macrophages” are associated with “smokers bronchiolitis” or “respiratory bronchiolitis” and were characterized by the presence of macrophages containing dusty brown non-hemosiderin pigment in tubular airways (Couture and Colby, 2003). Pneumocyte hyperplasia was scared based on an increase in the size and shape of alveolar pneumocytes. The pathological scores ranged from 0 to 2+, and there were few 2+ scores. For that reason, the scores in each mouse were converted to ordinal data: the simple presence or absence of the pathological finding, and the presence (1) or absence (0) of each particular pathological finding was compared among groups using a non-parametric, One-Way ANOVA (Kruskal–Wallis Test).

### Histomorphometric analysis

Mean Linear Intercept (*L*_*M*_) is a reflection of the mean air space diameter (Hsia et al., 2010). This variable was chosen to give additional information about alveolar abnormalities, observed as air space enlargement (Heemskerk-Gerritsen et al., 1996; Robbesom et al., 2003; Jacob et al., 2009). Images (JPEG) of histological sections of the glutaraldehyde fixed lungs were taken using a Diagnostic Instruments, Inc., 11.2 Color Mosaic digital camera (at 1600 × 1200 pixel resolution) using the SPOT Basic software, a 40× objective lens, and a 10× eyepiece on an Olympus BX50 microscope. We calculated that 2.764 pixels at this magnification were equal to one micron. From each lung section, ten random pictures were analyzed.

In Matlab® (a high-level technical computing language), each image was automatically separated into two color planes (tissue and white space) using K-means clustering (partitioning algorithm). White spaces with areas of less than 500 pixels were excluded from this analysis so as to avoid measuring vessels and stray white pixels. In parallel horizontal lines 25 microns (69 pixels) apart, transitions from airspace to tissue were automatically counted, as was the total distance measured (28,800 pixels, or 10,420 microns). The mean linear intercept (*L*_*M*_) in each image was then calculated in microns according to:

$$L_{M}=\frac{\left(N\times l\right)}{\sum I}$$

where *N* is the number of times the line is placed on the image, *l* is the length of each line and ∑*I* is the total number of transitions from airspace to tissue measured.

### Quantitative PCR

For gene expression studies in the lung, we selected genes known to be involved in development of emphysema. Tissues for mRNA analysis were prepared from excised lungs that were placed in RNAlater® (Qiagen, Valencia, CA) and frozen at -80 C. RNA was extracted from each sample using RNeasy® (Qiagen, Valencia, CA). One microgram of RNA was reverse transcribed to cDNA using an iScript™ kit (Bio-Rad, Hercules, CA) and amplified by real time PCR using SYBR green master mix (Bio-Rad, Hercules, CA). Gene expression levels were normalized to mouse housekeeping gene RPL13a (MHK) expression. The primer sets used are shown in Table 1. Quantification was determined using the software *Opticon Monitor* software version 3.1.

**Table 1:** **Primers used for RT-PCR**.

**Gene**	**Primers**
TIMP1	F 5-CCGCAGTGAAGAGTTTCTCA-3
	R 5-TCACTCTCCAGTTTGCAA GG-3
MHK-RPL13a	F 5-ATGACAAGAAAAAGCGGATG-3
	R 5-CTTTTCTGCCTGTTTCCGTA-3
MMP9	F 5-CTCACTCACTGTGGTTGCTG-3
	R 5-TGGTTATCCTTCCTGGATCA-3
A1AT	F 5-TTCCAACACCTCCTCCAAAC-3
	R 5-CACTTTCTTGGCCTCCTCTG-3

F, forward; R, reverse; TIMP, tissue inhibitor of metalloproteinases; MMP, matrix metallopeptidase; A1AT, alpha 1-antitrypsin or α1-antitrypsin.

## Results

### Vitamin D levels are decreased in VDD mice

**Figure 1:**
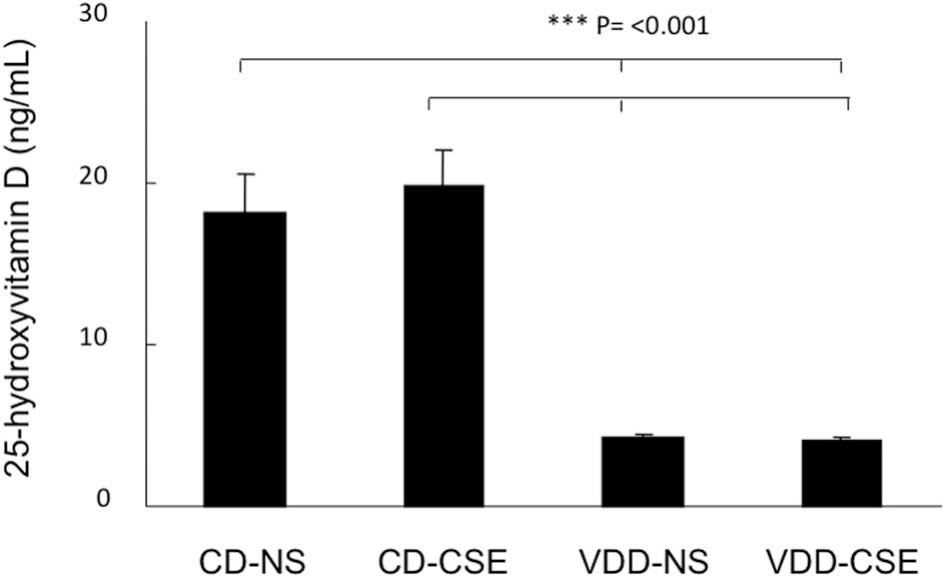
**25-hydroxyvitamin D levels in the four treatment groups at the end of the experimental period.** Values shown reflect means ± SEM of 19–23 mice per group. Levels of 25-hydroxyvitamin D in mice fed control diets were significantly different than mice fed a vitamin D deficient diet (*p* = 0.001).

To confirm that the vitamin D deficient diet resulted in low vitamin D levels, we measured vitamin D levels in all mice. All vitamin D levels were measured at the conclusion of each study at the time of sacrifice. The mean serum level of 25-hydroxyvitamin D3 in CD-NS mice was 18.2 ± 11.2 ng/mL and in CD-CSE it was 19.9 ± 9.8 ng/mL. Mice on the vitamin D deficient diet had levels of <5 ng/mL in both NS and CSE conditions (Figure 1). Thus, levels of 25-hydroxyvitamin D in mice fed control diets were significantly greater than 25-hydroxyvitamin D levels in mice fed a vitamin D deficient diet (*p* = 0.001).

### Pathology

**Figure 2:**
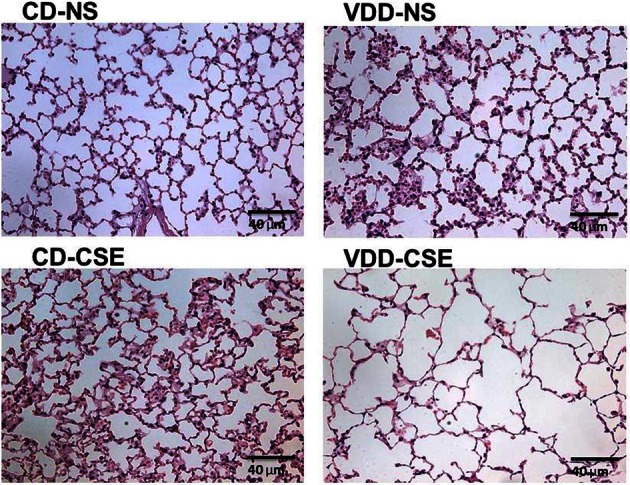
**Normal and emphysema-like features in hematoxylin and eosin (H&E) stained sections of lung representative of lung sections from each treatment group.** All images are 40× enlargements.

In order to explore the effect of CSE on lung architecture and the frequency of pathological findings within the lung, the histological slides were examined in a blinded fashion. Representative sections are shown in Figure 2 and summary statistics, based on the presence or absence of specific pathological features in each treatment group, are shown in Table 2. Statistically significant differences were noted between treatment groups in the occurrence of pigmented macrophages within small airways in 8 of 11 of the VDD-CSE group and in 5 of 11 of the CD-CSE mice (*p* < 0.05 for both groups compared to control, CD-NS animals). No pigmented macrophages were noted in sections of lung of the mice breathing filtered room air. There were only rare instances of acute neutrophilic inflammation in any of the specimens, but chronic inflammation, indicated by the presence of lymphocytic inflammatory infiltrates, was seen sporadically in all groups of mice. However, none of the other pathological features differed significantly among groups.

**Table 2:** **Histological review of mouse lungs**.

	**Airway inflammation (%)**	**Parenchymal inflammation (%)**	**Pigmented macrophages (%)**	**Pneumocyte hyperplasia (%)**
CD-NS	44	78	0	22
VDD-NS	55	73	18	45
CD-CSE	0	67	75^*^	0
VDD-CSE	36	55	91^*^	18

Sections of H&E-stained mouse lung were reviewed for the presence/absence of the four histologic features indicated in Table 2. The frequency of occurrence of these features is represented as percentages of animals within each treatment group in which the histological feature was observed. All of the inflammation in these cases was chronic (lymphocytic); there were no cases that showed acute (neutrophil) cells.

* Indicates a significant increase in a particular feature compared to control animals (CD-NS) at the p < 0.05 level.

### Morphometric analysis reveals enlargement of air spaces in VDD-CSE mice

**Figure 3:**
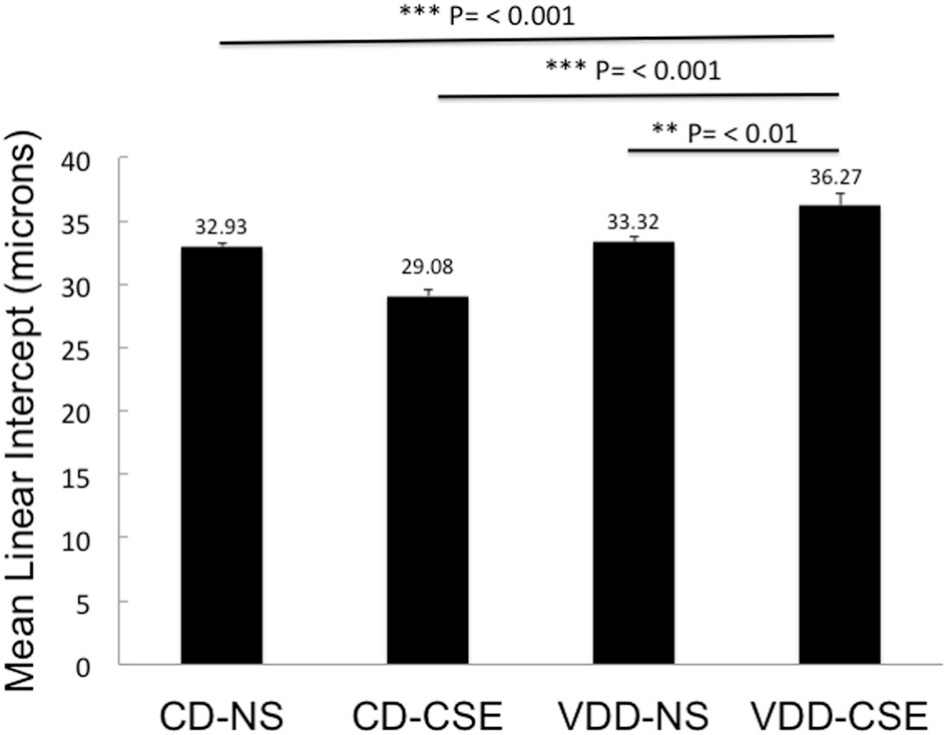
**Mean Linear Intercept (*L*_*M*_) in experimental groups.** Levels shown reflect means ± SEM. *L*_*M*_ is significantly longer in VDD-CSE mice compared to all other treatment groups and is consistent with emphysema. Data shown are based on analyses of 10 images from each lung section of each of eleven mice in each of the four treatment groups (440 total images analyzed).

Morphological measurements represent a useful, unbiased method to characterize emphysematous lesions in a semi-quantitative manner (Robbesom et al., 2003). Enlargement of air spaces was evaluated by the Mean Linear Intercept (*L*_*M*_) measurement technique (Thurlbeck, 1967). Based on analysis of 10 images from each lung section of each of eleven mice in each of the four treatment groups (440 total images analyzed), mice with VDD-CSE treatments had significantly longer mean linear intercept (*L*_*M*_) distance (36.27μ ± 0.88) compared to animals in the other three groups (Figure 3). CD-NS MLI was 32.93 μ (±0.37, *p* = 0.0006). VDD-NS was 33.32 μ (±0.47, *p* = 0.0037) and CD-CSE was 29.08 μ (±0.487, *p* = 0.00001). Thus, VDD-CSE mice had the greatest morphometric air space enlargement, consistent with a contribution of vitamin D deficiency to the development of emphysema.

It is surprising that the CD-CSE animals had, on average, a normal *L*_*M*_. The analysis was, however, confounded by the co-occurrence of accumulations of macrophages. As shown in Table 1, there was a striking increase in the number of animals with increased alveolar macrophages in the airspace among the smoke exposed animals. The measurement of *L*_*M*_ was automated to preclude bias and measured airspace size—not inter-septal distances across alveoli (which is consistent with the ATS Standards for Quantitative Assessment of Lung Structure; (Hsia et al., 2010), and these standardized measurement methods of are specifically meant to exclude changes in septal thickens). Moreover, others have noted that smoke exposure in mice may increase macrophage numbers (Hirama et al., 2007). Thus, increased macrophage accumulation in the alveolar airspace in the CD-CSE animals may have reduced the measured *L*_*M*_ despite the presence of alveolar enlargement shown in some regions of the lung (Figure 2, CD-CSE example).

### Mice exposed to cigarette smoke have altered respiratory mechanics

**Figure 4:**
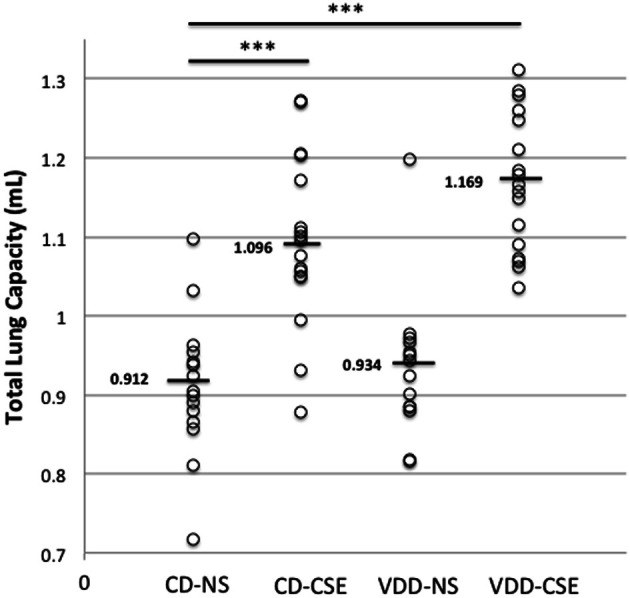
**Total lung capacity (TLC) in experimental groups.** Over the course of three separate experiments, total lung capacity was determined by *flexi*Vent™ in 63 mice. Each point represents the data from one mouse with 14 to 17 mice per treatment group. Horizontal bars mark medians. A Two-Way ANOVA indicated that the mean TLC in the VDD-NS group and the CD-CSE group was significantly greater than the mean TLC in the control group (CD-NS) (indicated by ^***^ in the figure; *p* = 0.001).

Respiratory mechanics are used in COPD and emphysema as an measure of disease severity and progression. To determine if exposure to cigarette smoke and a vitamin D deficient diet influenced lung function, 63 mice were subjected to analysis of respiratory mechanics using a *flexi*Vent™ ventilator. Using the forced oscillation methods described above, static compliance increased significantly in CD-CSE and the VDD-CSE as compared to controls (*P* < 0.05 for both groups; data not shown). However, there was no significant difference in compliance between VDD-CSE mice and CD-CSE mice; compliance was similarly elevated in both groups. Pressure-volume loops were used to estimate Total Lung Capacity (TLC) (Vanoirbeek et al., 2010), an important indicator of the severity and progression of emphysema (Pauls et al., 2010). Consistent with the measurements of compliance, the TLC was also increased in both CD-CSE and VDD-CSE mice compared to the control animals (CD-NS) (Figure 4). The TLC and static compliance were elevated in the VDD-CSE mice, and animals in this treatment group had the longest average *L*_*M*_. Thus, the measurements of respiratory mechanics were consistent with the morphometric analysis of alveolar diameters in VDD-CSE mice. The same was not true of the CD-CSE mice in whom compliance and TLC were significantly increased compared to CD-NS animals, but *L*_*M*_ was not increased. As noted above, the presence of increased numbers of pigmented macrophages may have confounded the measurements of *L*_*M*_ in the CD-CSE mice, but it is also true that changes in alveolar dimensions may lag changes in respiratory mechanics in cigarette smoke exposed mice (Rinaldi et al., 2012).

### mRNA expression in lung tissues is altered in VDD-CSE mice

**Figure 5:**
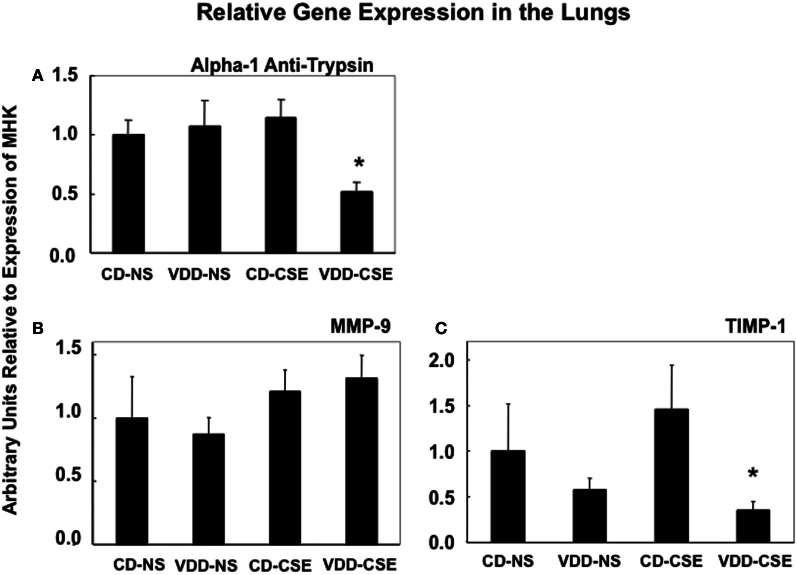
**Bars represent the ratio ± SEM of expression of genes to expression of the MHK housekeeping gene from whole lung in 4–8 mice from each treatment group. (A)** Expression of A1AT is significantly different in VDD-CSE mice from control mice (CD-NS) (*p* = 0.0060), from CSE (*p* = 0.0019), and from VDD mice (*p* = 0.0290). **(B)** While mean values of MMP-9 expression suggest an increase in CSE and VDD-CSE treated mouse lungs, expression of MMP-9 was not significantly different when comparing various treatment groups. **(C)** Expression of TIMP-1 was significantly different in VDD-CSE mice from CSE group (*p* = 0.0448).

**Figure 6:**
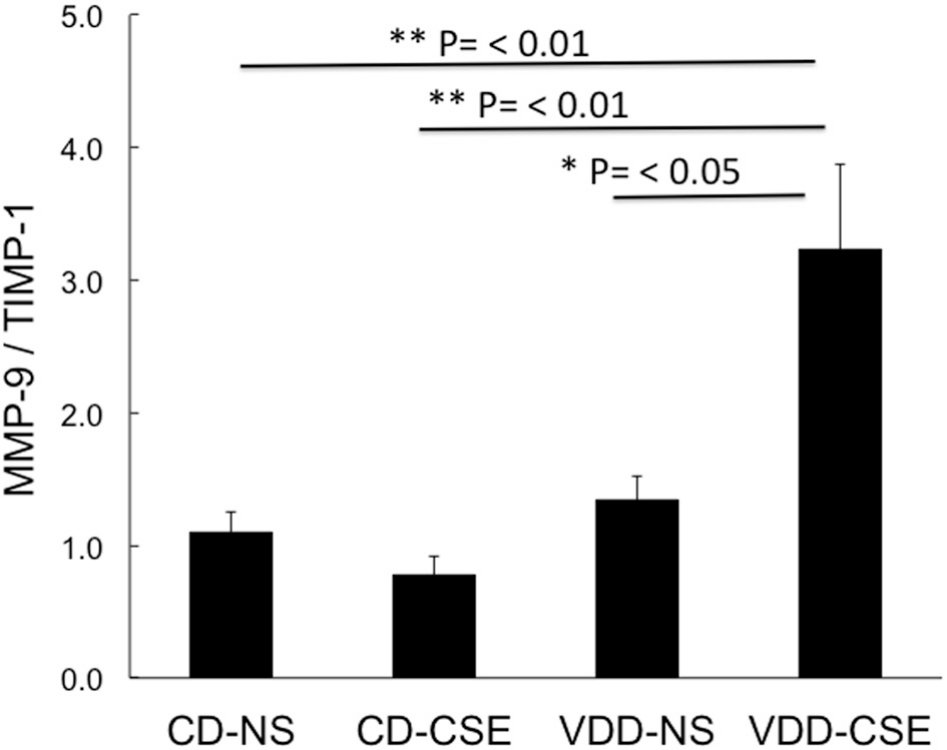
**Bars represent the ratio ± SEM of expression of MMP-9 divided by TIMP-1 in each animal studied.** Both TIMP-1 and MMP-9 are standardized to expression of the MHK housekeeping gene from whole lung from four to seven mice from each treatment group. Expression of the TIMP-1/MMP-9 ratio was significantly different comparing VDD to VDD-CSE (*p* = 0.05) and CD-NS and CD-CSE to VDD-CSE (*p* = 0.01).

Proteases and their inhibitors are involved in tissue damage in emphysema. To begin to address the mechanisms of increased emphysema in the VDD-CSE mice, we looked at genes previously associated with COPD (Kang et al., 2003; Mercer et al., 2005; Tuder et al., 2010). We did quantitative real time PCR analysis of relative mRNA expression in the four treatment groups comparing gene expression of alpha-1 antitrypsin (A1AT), MMP9, and TIMP-1, to the expression of the mouse housekeeping gene RPL13a (MHK). While neither the CD-CSE nor the VDD-NS group mice had a significant change in A1AT, when the two treatments were combined in the VDD-CSE mice, lung expression level of this gene, crucial in protecting the lung against development of emphysema, was significantly decreased (Figure 5A). MMPs and their inhibitors, TIMPs, are also important in the pathogenesis of emphysema (Smolonska et al., 2009). While we found a non-significant trend toward increased expression of MMP-9 when comparing CD-NS to VDD-CSE (Figure 5B), analyses revealed a significant drop in expression of its cognate inhibitor, TIMP-1 (Mercer et al., 2005) only in the VDD-CSE group (Figure 5C). The ratio of MMP-9 to TIMP-1 was significantly different comparing VDD to VDD-CSE (*p* = 0.05) and CD-NS and CD-CSE to VDD-CSE (*p* = 0.01) (Figure 6). At the expression level, the increase in MMP-9, the decrease in TIMP-1 and the decrease in A1AT suggest that a significant imbalance in favor of proteolysis existed in the VDD-CSE mice.

## Discussion

Lifespan, period of exposure required to develop disease, as well as heterogeneity of lifestyle and co-morbid factors preclude straightforward examination of the contribution of vitamin D deficiency to development of emphysema in humans. For this reason we developed a new, pragmatic model for the study of emphysema by combining Vitamin D deficiency and cigarette smoke exposure in mice. To our knowledge this is the first model to combine dietary vitamin D deficiency and smoke exposure. We demonstrated an effect of vitamin D deficiency and cigarette smoke exposure on the mean linear intercept, the total lung capacity and lung compliance and a shift in the protease/antiprotease balance within the lung that favored proteolysis in this model in the vitamin D deficient animals. Our outcomes are consistent with epidemiological associations between the low Vitamin D levels, cigarette smoke exposure and COPD. These data support a role for vitamin D deficiency in development of emphysema, and our murine model provides a platform to study the effect of cigarette exposure and vitamin D deficiency on the immunopathogenesis of this common debilitating disease.

The importance of having a model in which to study the relationship of vitamin D deficiency to cigarette smoke induced lung damage is supported by a number of observations. Vitamin D deficiency occurs frequently in COPD, and the severity of vitamin D deficiency correlates with the severity of COPD (Janssens et al., 2009, 2010). However, whether vitamin D deficiency is a consequence of COPD, a comorbid factor in COPD, or a contributing causal risk factor in the pathogenesis of COPD cannot be fully determined from epidemiological data. Vitamin D deficiency could be a consequence of COPD since patients with COPD spend more time indoors when compared to people of the same age and location without COPD (Donaldson et al., 2005). In addition, aging skin (both chronological and from smoking) and glucocorticoids (which are prescribed frequently to patients with COPD and may increase vitamin D metabolism) can compound vitamin D deficiency in COPD patients (Hughes and Norton, 2009). Alternatively, vitamin D deficiency could result in skeletal changes (Franco et al., 2009), muscle weakness (Hopkinson et al., 2008), or propensity to infectious exacerbations (Ginde et al., 2009); all of which are comorbid factors in COPD. Another possibility is that vitamin D deficiency, through its effects on innate and adaptive immunity (Adams and Hewison, 2008; Hewison, 2010; Miller and Gallo, 2010), could serve as a contributing causal, risk factor for COPD, a disease mediated at least in part by inflammatory cell-derived proteases (Churg et al., 2008).

This model of cigarette smoke exposure was designed to address whether low vitamin D levels can cooperate with cigarette smoke exposure in the pathogenesis of emphysema. We demonstrated an increased incidence of smoke-induced lung disease in vitamin D deficient-cigarette smoke exposed mice compared to control diet-cigarette smoke exposed mice. Specifically, morphometric assessment of alveolar dimensions, using the average alveolar size as estimated by the mean linear intercept (*L*_*M*_), was significantly increased in the VDD-CSE group, which is consistent with the occurrence of emphysema. The *L*_*M*_ distance in the CD-CSE mice was not increased, which was contrary to our expectations. The *L*_*M*_, as we measured it and as is recommended by the American Thoracic Society, measures the airspace dimensions only. Images of representative lung sections (see Figure 2, bottom left) raise the possibility that the relatively smaller *L*_*M*_ in the CD-CSE mice reflects both increased numbers of macrophages (Hirama et al., 2007; Bodas et al., 2011) and less prominent or slower developing emphysema in mice exposed to cigarette smoke (CD-CSE) for only 16 weeks, as compared to the mice with combined vitamin D deficiency and cigarette smoke exposure. The presence of increased macrophages in the airspace of the CD-CSE animals, which is typical of cigarette smoke exposed animals and humans, may have caused us to underestimate the “true” *L*_*M*_ in the CD-CSE mice. This latter explanation is consistent with our *flexi*Vent™ data, which revealed increased TLC and lung compliance in both CD-CSE and VDD-CSE groups. The asymmetrical deposition of macrophages within individual alveoli may not have altered the intrinsic elastic characteristics of the lung, which make a major contribution to the compliance of the respiratory system and to the TLC, but may have diminished the *L*_*M*_ of the alveoli with particularly heavy infiltration of macrophages.

It seems more likely to us, however, that the morphometric changes in lung associated with cigarette smoke exposure simply take longer to develop than the changes in respiratory mechanics (Rinaldi et al., 2012). Emphysematous changes in lung morphology usually occur in mice only after 26 weeks of cigarette smoke exposure (Shapiro et al., 2003). Thus, we found the highest TLC, the highest compliance and the largest *L*_*M*_ after only 16 weeks of cigarette smoke exposure in the vitamin D deficient animals. The next highest average TLC and compliance measurements occurred in the CD-CSE animals, though *L*_*M*_ was not similarly increased. Thus, adding vitamin D deficiency to cigarette smoke exposure may have accelerated the development of the pathological changes typical of emphysema so that the VDD-CSE group had pathological evidence of emphysema after only 16 weeks of cigarette smoke exposure; whereas previous investigators found that pathological evidence of emphysema appeared in non-vitamin D deficient mice only after 26 weeks of cigarette exposure even though changes in lung mechanics occurred after 12 weeks of cigarette smoke exposure (just as we found in the vitamin D replete animals after 16 weeks of cigarette smoke exposure) (Rinaldi et al., 2012). The CD-CSE mice were, in our view, developing emphysema (lung compliance and TLC were increased and there were focal areas of emphysema; see Figure 2), but the morphometric hallmark of emphysema (an increased *L*_*M*_) had not developed after only 16 weeks of cigarette smoke exposure. The possibility of accelerated emphysema formation when vitamin D was deficient is also supported by the greater increase in expression of proteolytic enzymes compared to antiproteolytic factors in the VDD-CSE mice compared to the mice exposed to either vitamin D deficiency or cigarette smoke alone.

Vitamin D is a steroidal hormone produced mainly from precursors within the skin via the action of ultraviolet B and available, to a lesser extent, from dietary sources. To define our model, mice on control diets were fed standard levels of vitamin D found in commercial mouse chow; in contrast the mice on VDD diet had no exogenous source of vitamin D. Vitamin D is recognized as an important hormone that controls genes with a multitude of extra-skeletal functions through its actions as a transcription factor (Bischoff-Ferrari, 2010; Holick, 2010) including control of the expression of a number of genes associated with innate immunity (Adams et al., 2007, 2009). Infectious exacerbations are increasingly frequent in the late stages of COPD in humans and vitamin D insufficiency or deficiency could contribute to COPD pathogenesis by diminishing protection against lung infection. Ongoing experiments are addressing this hypothesis.

To begin to address putative mechanisms whereby vitamin D deficiency might contribute to our observed findings, we looked at enzymes associated with lung remodeling and at TIMP-1, a gene that we noted in early experiments to be altered by cigarette smoke. MMPs have an important role in the breakdown of ECM and may serve as markers of tissue damage in smoking related lung diseases (Elkington and Friedland, 2006; Ilumets et al., 2007; Louhelainen et al., 2009). TIMP-1 is a specific inhibitor of MMP-9; low levels of TIMP-1 relative to MMP-9 suggest a proteinase rich environment that would foster breakdown of the extracellular matrix. The MMP-9:TIMP-1 ratio is increased in sputum of COPD patients (Beeh et al., 2003) and plasma MMP-9 levels are inversely related to vitamin D levels (Timms et al., 2002). It is not surprising then that vitamin D suppresses the production of MMPs and enhances the level of tissue inhibitor of metalloproteinase-1 (TIMP-1) (Anand and Selvaraj, 2009). Thus, our data indicating an increase in MMP-9: TIMP-1 ratio in vitamin D deficient smoke-exposed mice provides a mechanism whereby vitamin D deficiency can influence and accelerate the development of emphysema. In keeping with a role for vitamin D deficiency in protease-antiprotease imbalance, we also found significantly reduced A1AT levels in vitamin D-deficient smoke-exposed mice. Alpha-1 antitrypsin deficiency (AATD) confers susceptibility to emphysema, and polymorphisms in vitamin D-binding protein (VDBP; also known as Gc-globulin) have been linked to AATD (Chishimba et al., 2010).

In this study, designed to address whether low vitamin D levels contribute directly to aspects of COPD and emphysema pathogenesis, we found increased emphysema (increased alveolar *L*_*M*_, increased static compliance and increased TLC and lung compliance in the VDD-CSE treatment group) and changes in gene expression consistent with enhanced proteolytic activity in the lungs of vitamin D deficient-cigarette smoke exposed mice after a relatively short duration of exposure to cigarette smoke. Moreover, our data suggest that a protease-antiprotease imbalance, which developed only after combined exposure to cigarette smoke and a vitamin D deficient diet, may have accelerated the development of emphysema so that the changes in lung mechanics and lung pathology typical of COPD in mice developed within 16 weeks of cigarette smoke exposure in the vitamin D deficient animals rather than the usual 26 week exposure required to generate all of these changes in previous studies in vitamin D replete, cigarette smoke exposed mice (Shapiro et al., 2003; Rinaldi et al., 2012). In conclusion, this work establishes a potentially useful murine model to study the combined effects of vitamin D deficiency and cigarette smoke exposure in the development of emphysema. Ongoing studies are focusing on the interaction of vitamin D and cigarette smoke in respiratory tract innate immunity.

## Author contributions

Crane-Godreau—hypotheses, experimental design and organization, mouse smoke exposure, manuscript; Jukowski, Dechen and Ryu—molecular studies, mouse smoke exposure; Giustini and Hoopes—morphometrics; Lee—Vitamin D analysis; Black and Ratcliffe-pathology; Bessette—lung mechanics; Fiering—molecular analysis; Kelly—lung mechanics, design and manuscript, Leiter—statistics, manuscript.

### Conflict of interest statement

The authors declare that the research was conducted in the absence of any commercial or financial relationships that could be construed as a potential conflict of interest.

## Support

Flight Attendant Medical Research Institute (Crane-Godreau YCSA 052360 and CIA 103050), American Cancer Society (Crane-Godreau, ACS #IRG-82-003-24), Veteran's Administration Merit Review Award (Kelly Merit Award 2009-13), ZRT Laboratory (Donation of some 25-hydroxyvitamin D testing). The funders had no role in study design, data collection and analysis, decision to publish, or preparation of the manuscript.
